# A review of long non-coding RNAs in ankylosing spondylitis: pathogenesis, clinical assessment, and therapeutic targets

**DOI:** 10.3389/fcell.2024.1362476

**Published:** 2024-03-25

**Authors:** Hanji Wang, Chengxian Yang, Ge Li, Boning Wang, Longtao Qi, Yu Wang

**Affiliations:** ^1^ Department of Orthopaedics, Peking University First Hospital, Beijing, China; ^2^ Department of Endocrinology, Peking University First Hospital, Beijing, China

**Keywords:** ankylosing spondylitis, long non-coding RNA, pathogenesis, biomarker, therapy

## Abstract

Ankylosing spondylitis (AS) is a chronic immune-mediated type of inflammatory arthritis characterized by inflammation, bone erosion, and stiffness of the spine and sacroiliac joints. Despite great efforts put into the investigation of the disease, the pathogenesis of AS remains unclear, posing challenges in identifying ideal targets for diagnosis and treatment. To enhance our understanding of AS, an increasing number of studies have been conducted. Some of these studies reveal that long non-coding RNAs (lncRNAs) play crucial roles in the etiology of AS. Some certain lncRNAs influence the development of AS by regulating inflammatory responses, autophagy, apoptosis, and adipogenesis, as well as the proliferation and differentiation of cells. Additionally, some lncRNAs demonstrate potential as biomarkers, aiding in monitoring disease progression and predicting prognosis. In this review, we summarize recent studies concerning lncRNAs in AS to elucidate the underlying mechanisms in which lncRNAs are involved and their potential values as biomarkers for disease assessment and druggable targets for therapy.

## Introduction

Ankylosing spondylitis (AS) is an autoimmune disease characterized by pathological osteogenesis and systemic inflammation, primarily affecting ligaments, tendon attachment points, and the axial skeleton. The clinical features of AS mainly include back pain, spinal limitations, and various extra-articular symptoms (Braun and Sieper, 2007; Taurog et al., 2016). AS can affect individuals of any age, with a prevalence of 0.03%–0.09%. However, AS is most commonly diagnosed in individuals between the ages of 20 and 30, with a male-to-female ratio of approximately 2:1 (Braun and Sieper, 2007; DeLay et al., 2009). The condition can cause severe stiffness and even fusion of the spine and pelvis, leading to a significantly decreased quality of life. Various studies have explored the pathogenesis of AS, revealing involvement in some processes such as HLA-B27 misfolding, bacterial infection, macrophage activation, certain cytokines, and autophagy (Braun et al., 1995; Rashid and Ebringer, 2007; DeLay et al., 2009; Gaston et al., 2011; Ciccia et al., 2014; Hunter and Jones, 2015; Ciccia and Haroon, 2016). Quite a number of things have been done to explore the target cells playing important roles in the development of AS. For example, the mesenchymal stem cells (MSCs), was reported that may resulting in AS in the conditions of oxidative stress-mediated mitochondrial dysfunction (Ye et al., 2020) and TNF-α-mediated m6A modification of ELMO1 (Xie et al., 2021). Likewise, ankylosing spondylitis fibroblasts (ASFs) can also influence the progression of AS by promoting joint remodeling, affecting fat enrichment and activating as myofibroblasts to form extracellular matrix independent of inflammation (Liu et al., 2023). Given that dysregulation of inflammatory responses are primary manifestations of AS, treatment strategies primarily focus on non-steroidal anti-inflammatory drugs and biologics, including tumor necrosis factor inhibitors and interleukin-17 inhibitors (Deodhar et al., 2020; Tam et al., 2022). Surgical intervention becomes inevitable for individuals experiencing serious deformities and high disease activity (Liu et al., 2022). The treatment of AS remains a clinical challenge. Consequently, it is of great significance to identify new therapeutic targets and ideal biomarkers to monitor AS activity.

Long non-coding RNAs (lncRNAs) have been demonstrated to regulate gene expression at multiple levels by acting as competitive endogenous RNAs and interacting with microRNAs (miRNAs) to inhibit the expression of target genes (Tay et al., 2014; Lalevée and Feil, 2015; Kwok and Tay, 2017). The expanding body of research reveals that lncRNAs play pivotal roles in various biological processes, including brain development, embryonic development, histological differentiation, organogenesis, cell proliferation, tumor metastasis, cell growth, and cell differentiation (Pollard et al., 2006; Beniaminov et al., 2008; Mercer et al., 2009; Gupta et al., 2010; Prensner et al., 2013). In the research area of immune-related lncRNAs, investigations have unveiled that these lncRNAs are strategically located adjacent to, or partly overlapping with, the 5′end or 3′end of protein-coding genes involved in immune responses. This suggests that lncRNAs could exert influence on inflammatory responses by acting as cis-acting or trans-acting factors (Brosius, 2005; Derrien et al., 2012). Emerging evidence supports that dysregulation of lncRNAs plays a pivotal role in the pathogenesis of AS. In this comprehensive review, we provide a summary of relevant lncRNAs associated with AS, elucidating their involvement in pathophysiological mechanisms. Additionally, we explore their potential utility as diagnostic or prognostic markers and examine their viability as druggable targets for the treatment of AS.

## LncRNAs involved in the pathogenesis of AS

### Inflammatory responses of AS

Numerous studies have confirmed the significant role of immune-mediated inflammatory responses in the pathogenesis of AS, primarily through pathways involving TNF, IL-23, and others (Brown et al., 2016). Dysregulation of inflammatory cytokines has been extensively identified across various sample types. Considering that lncRNAs can influence the transcription of inflammatory cytokines through interactions with miRNAs and other mechanisms, substantial efforts have been undertaken to investigate the involvement of lncRNAs in the inflammation associated with AS.

In a study conducted by Zhang et al., it was observed that the lncRNA H19 exhibited a significant upregulation in AS patients compared to healthy controls (Zhang et al., 2020). This finding was further corroborated by another study conducted by Marwa M. Esawy et al. (Esawy et al., 2023). In the experiment conducted by Zhang et al., the knockdown of H19 downregulated vitamin D receptor (VDR) and miR-675-5p expressions while promoting miR-22-5p expression in peripheral blood mononuclear cells (PBMCs). Conversely, the overexpression of H19 led to the inhibition of miR-22-5p expression and promotion of IL-17 and VDR expression. As demonstrated, H19 regulated inflammation by influencing the VDR-IL-23/17A signaling axis through interactions with miR-675-5p and miR-22-5p.

In the study conducted by Gai X et al., it was established that the plasma levels of NF-κb interacting lncRNA (NKILA) and transforming growth factor-beta 1 (TGF-β1) were significantly elevated in the fasting blood of the active disease group compared to both the inactive disease group and healthy controls (Gai and Li, 2019). Consistently, previous studies have confirmed the pivotal role of TGF-β in the development and progression of AS (Vaez et al., 2017), given that the expression of TGF-β1 is positively correlated with disease activity. These results suggest that NKILA may influence the progression of AS by interacting with TGF-β1.

In an earlier study, the researchers used high-throughput sequencing and bioinformatics analysis to identify the lncRNA NONHSAT227927.1 as a significant player in the inflammatory processes of AS through the application of (Wang et al., 2021). Subsequent experiments revealed that the levels of IL-17 and IL-23 notably increased and the IL-10 level decreased with the overexpression of NONHSAT227927.1. Conversely, silencing NONHSAT227927.1 exhibited opposite trends. These findings suggest that NONHSAT227927.1 may indeed be a pivotal lncRNA influencing the progression of AS (Ding et al., 2023). In a recent study conducted by Yu HC et al., it was observed that the levels of lncRNA LOC645166 in T cells of AS patients were diminished (Yu et al., 2021). The upregulation of LOC645166 expression can suppress the IL-23p19 expression and JAK2/STAT-3 signaling of Jurkat cells in response to treatment with phorbol 12-myristate 13-acetate (PMA). LOC645166 can suppress recruitment of the IκB kinase (IKK) complex to K63-linked polyubiquitin chains and diminish IKK2 activation, resulting in the downregulation of nuclear factor kappa-B(NF-κB) activation.

Beyond experimental studies, numerous researchers have dedicated efforts to bioinformatics analyses. In a study by Li YX et al., 147 lncRNAs, including 107 upregulated and 40 downregulated lncRNAs, were identified as differentially expressed in Toll-like receptor 4 (TLR4)-primed mesenchymal stem cells (MSCs) compared to unprimed MSCs from AS patients (Li et al., 2021). Gene Ontology and Kyoto Encyclopedia of Genes and Genomes (KEGG) analyses revealed a high association of differentially expressed mRNAs and lncRNAs with the inflammatory response, such as the TNF signaling pathway and the NF-κB signaling pathway. Similarly, a total of 435 mRNAs and 45 lncRNAs were found to be downregulated in the AS group, with 284 mRNAs and 114 lncRNAs upregulated (Li et al., 2022). KEGG enrichment analysis based on trans-target genes indicated that differentially expressed lncRNAs were enriched in pathways such as the B cell receptor signaling pathway, TNF signaling pathway, NF-κB signaling pathway, etc.

### Cell proliferation and differentiation in AS

The lncRNA HOXA transcript at the distal tip (HOTTIP) is transcribed from the 5′tip of the HOXA cluster (Zhao et al., 2018). HOTTIP has been implicated in the pathogenesis of osteosarcoma, endochondral ossification, and osteoarthritic progression (Kim et al., 2013; Li et al., 2015). In an experiment conducted by Wei L et al., elevated levels of HOTTIP were detected, while miR-30b-3p was reduced in AS synovial tissues and AS fibroblast-like synoviocytes (ASFLSs) in mouse models (Wei et al., 2023). The study further suggested that overexpressed miR-30b-3p or inhibited HOTTIP hindered the proliferation and differentiation of ASFLSs by suppressing phosphoglycerate kinase 1(PGK1), thereby restraining AS development.

Ma et al. demonstrated that upregulated expression of lncRNA MEG3 (maternally expressed 3) could result in the sponging of let-7i, which led to the restoration of sclerostin expression and repressed the release of pro-inflammatory cytokine in AS fibroblasts (Ma et al., 2020). A study by Liu C et al. elucidated that MEG3 could competitively bind to miR-125a-5p to upregulate the expression of TNF alpha-induced protein 3 (TNFAIP3), thereby repressing the Wnt/β-catenin pathway and downregulating the osteogenic differentiation of MSCs (Liu et al., 2023). In proteoglycan-induced AS mouse models, MEG3 also reduced the osteogenic activity of MSCs, inhibiting AS progression through the miR-125a-5p/TNFAIP3/Wnt/β-catenin axis. Moreover, earlier studies demonstrated that lncRNAs such as lnc-ZNF354A-1, lnc-FRG2C-3, lnc-USP50-2, and lnc-LIN54-1 might participate in the abnormal osteogenic differentiation of MSCs from patients with AS (Xie et al., 2016).

### Autophagy and apoptosis of cells in AS

Autophagy has recently emerged as a regulatory mechanism in immunity and inflammation, with several studies suggesting its potential involvement in the development of AS (Neerinckx et al., 2014; Park et al., 2017; Wang et al., 2017). In a study conducted by Tan M et al., it was revealed that the lncRNA growth arrest-specific 5 (GAS5) was downregulated in the PBMCs of AS patients compared to healthy controls. Moreover, GAS5 exhibited a positive correlation with the expression of key autophagy-related genes, including, Beclin1, autophagy-related gene (ATG)3, ATG5, ATG12, and ATG16. These findings strongly support the hypothesis that lncRNA GAS5 plays a role in the pathogenesis of AS by regulating autophagy (Tan et al., 2020).

A specific lncRNA, highly upregulated in liver cancer (HULC), has been investigated for its functions and related mechanisms in AS (Yi et al., 2023). Their research demonstrated that the silencing of HULC promotes chondrocyte proliferation while reducing apoptosis and inflammation in AS. MiR-556-5p was identified as a downstream factor of HULC, and the induction of miR-556-5p protected chondrocytes from AS-induced damage. Yes-associated protein 1 (YAP1) was identified as a potential target gene of miR-556-5p, and notably, YAP1 overexpression counteracted the protective effects against AS induced by silenced HULC.

### Adipogenesis of ankylosing spondylitis mesenchymal stem cells

The increased adipogenesis observed in ankylosing spondylitis mesenchymal stem cells (ASMSCs) has the potential to induce fat metaplasia and influence the process of the formation new bone. Numerous lncRNAs have been documented to exert regulatory effects on the adipogenic differentiation process (Squillaro et al., 2020). In a study by Cen S et al., the analysis of ASMSCs during adipogenesis in comparison to human-derived mesenchymal stem cells revealed 137 upregulated and 126 downregulated lncRNAs (Cen et al., 2022). Notably, three of the top 10 differentially expressed lncRNAs (ENST00000429588.1, ENST00000400755.3, and ENST00000512300.1) exhibited significant co-expression with differentially expressed mRNAs. This suggests that these lncRNAs may represent crucial targets in the context of aberrant adipogenesis. However, more investigations are needed to explore their specific roles in the future.

### LncRNAs in the clinical evaluation of AS

The high sensitivity and responsiveness to dynamic changes in the disease underscore the promising potential of lncRNAs as biomarkers. Consequently, they hold the capability to facilitate the monitoring of disease activity, assess therapeutic efficacy, and provide insights into the prognosis AS.

### LncRNAs in synovial fluid

The expression of lncRNA-neighboring enhancer of FOXA2 (NEF) in the synovial fluid extracted from affected sites (38 cases of the joint between the base of the spine and pelvis and 22 cases of vertebrae in the lower back) was found to be higher than in the control groups (Han et al., 2022). Furthermore, a significantly lower recurrence rate of AS was observed in patients with low lncRNA-NEF levels compared to those with high lncRNA-NEF levels (hazard ratio = 2.266). Notably, lncRNA-NEF expression exhibited a significant correlation with Ankylosing spondylitis disease activity score (ASDAS), Bath Ankylosing Spondylitis Disease Activity Index (BASDAI), erythrocyte sedimentation rate (ESR), and C-reactive protein (CRP) levels (*p* < 0.05). Additionally, the expression of lncRNA-NEF was significantly downregulated in response to treatment with non-steroidal anti-inflammatory drugs (*p* < 0.01).

### LncRNAs in sacroiliac joint biopsies

The expression level of taurine-upregulated gene 1 (TUG1) was significantly downregulated in AS patients compared to healthy controls, as observed in open sacroiliac biopsies (Lan et al., 2018). Notably, individuals in the high expression group exhibited a prolonged hospitalization time and a higher rehospitalization rate (Liu et al., 2019). In a separate study, a similar trend was observed, with patients in the high expression group demonstrating an extended hospitalization time and an elevated rehospitalization rate. Conversely, the expression level of MEG3 in open sacroiliac joint biopsies was lower in AS patients than in healthy controls. Additionally, serum levels of MEG3 were not associated with age, sex, or alcohol/tobacco consumption of patients, but showed a close correlation with disease activity and disease duration. Patients with higher expression levels of MEG3 experienced a shorter hospitalization time and a lower rehospitalization rate.

In a bioinformatics analysis conducted by Li M et al., a total of 270 differentially expressed lncRNAs were identified, with 200 upregulated and 70 downregulated in AS patient samples (Li and Zhou, 2021). To further validate these findings, quantitative reverse transcription-polymerase chain reaction was employed to assess the expression of three upregulated and three downregulated lncRNAs in 15 AS patient samples relative to 15 control samples. The results indicated that the expression of NONHSAT118801.2 was positively correlated with ESR, BASDAI, and Bath Ankylosing Spondylitis Functional Index (BASFAI) levels in AS, while the expression of NONHSAT183847.1 was positively correlated with ESR, BASDAI, CRP, suggesting the potential value of these two lncRNAs in reflecting the progression of AS.

### LncRNAs in the blood samples

As blood is the most widely used sample in the clinics, many kinds of markers have been detected in the serum, PBMCs, and plasma. Notably, lncRNA-AK001085 was found to be significantly downregulated in AS patients compared to healthy controls (Li et al., 2017). Its expression was confirmed to be influenced by factors such as smoking, exercise level, and occupational activity level. Additionally, lncRNA-AK001085 levels showed a negative correlation with ESR, CRP, and ASDAS. Zhong H et al. found that upregulation of LINC00311 in the plasma distinguished AS patients from healthy controls with an AUC(Area Under Curve) of 0.9041 (Zhong and Zhong, 2019). Furthermore, BASDAI, ASDAS, CRP, and ESR were also significantly and positively correlated with plasma levels of LINC00311. LINC00311 levels in plasma were found to decrease after treatment.

Wang JX et al. conducted a bioinformatics analysis to screen several AS-related lncRNAs and extracted RNAs from PBMCs (Wang et al., 2022). To distinguish AS patients from healthy individuals, the results of diagnostic tests revealed that the AUC of linc00304, linc00926, and myocardial infarction-associated transcript (MIAT) lncRNA was 0.687, 0.664, and 0.623, respectively. Correlation analysis between the expression levels of target lncRNAs and AS activity indicators revealed that linc00304 expression was positively correlated with BASDAI, BASFI (Bath Ankylosing Spondylitis Functional Index), ESR, and CRP in AS. MIAT expression was positively correlated with BASFI, ESR, and CRP, while the expression level of linc00926 was only positively correlated with ESR. Similar to ESR and CRP, linc00304 was identified as independent risk factors for AS activity. They also found that lncRNA 326C3.7 was independently correlated with bone bridge formation, with an AUC of 0.739, which can aid in predicting the occurrence of bone bridge formation (Wang et al., 2021).

A recent study demonstrated that lncRNA intersectin 1–2 (lnc-ITSN1-2) expression in PBMCs was elevated in AS patients compared to controls (Li and Zhou, 2021). Lnc-ITSN1-2 expression was positively associated with CRP, BASDAI score, ASDAS, CRP, and IL-1β in patients with AS, but not with disease duration, ESR, BASFI score, total back pain score, TNF-α, IL-6, or IL-17A. During treatment, lnc-ITSN1-2 expression decreased in patients with AS. Reduced lnc-ITSN1-2 expression when patients were treated with TNF-α inhibitor correlated with treatment efficacy, which suggested that it can indicate the effect and prognosis.

### LncRNAs as the potential therapeutic targets in AS

As previously discussed, HULC has been identified as a potential influencer in the development of AS by regulating the expression of miR-556-5p and YAP1 (Yi et al., 2023). Additionally, elevated levels of HULC and YAP1, coupled with reduced miR-556-5p expression, were observed in the spinal cartilage of AS mice, indicating that HULC contributes to the pathological injury of spinal cartilage. These findings suggest that HULC could serve as a potential therapeutic target for AS.

Similarly, MEG3 has been identified as a promising therapeutic target due to its role as a negative regulator of inflammation. Except the miR-125a-5p/TNFAIP3/Wnt/β-catenin axis in MSCs and interaction with let-7i in FLSs elucidated formerly, Li Y et al. demonstrated that MEG3 suppressed inflammatory responses by modulating the expression of miR-146a, a key regulator of the innate immune response associated with the regulation of inflammatory reactions (Taganov et al., 2006; Bitar et al., 2019; Li et al., 2020). In a study by Li Y et al., the results showed significantly elevated expression of miR-146a in the serum of AS patients, showing a positive correlation with the levels of inflammatory cytokines (Li et al., 2020). Since MEG3 has been proved to play various roles in pathogenesis of AS especially by interacting with miR-146a, it is expected to provide a potential new means for the treatment of AS patients.

These findings hold significant implications for the exploration of targeted therapies for AS. However, the application of the lncRNAs as therapeutic targets still remains blank. The animal models in the experiment of Yi et al. provide the possibility that the intravenous injection of vector containing HULC may help to reverse the progression of cartilage histopathological injury.

## Conclusion and perspectives

**FIGURE 1:**
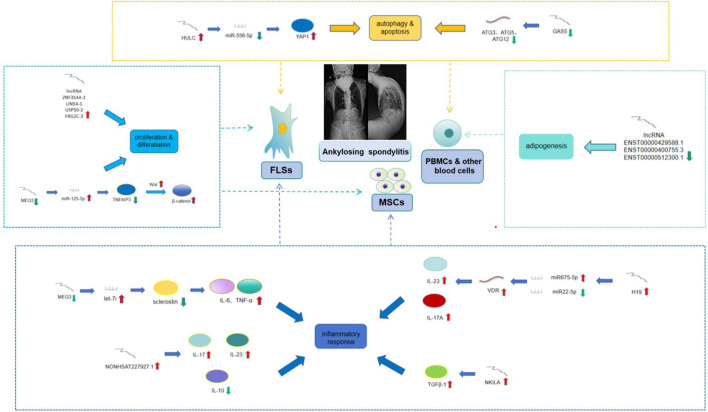
LncRNAs involved in the pathogenesis of AS and various target cells.

With an increasing number of researchers dedicating their efforts to this field, our understanding of the mechanisms involved in AS has advanced, incorporating epigenetic factors. Previous investigations have underscored the pivotal roles played by lncRNAs in various pathophysiological conditions (Table 1) through different kinds of target cells (Figure 1). It is anticipated that, through further mechanistic studies, additional functionally significant lncRNAs and associated pathways essential to AS pathogenesis and development will be elucidated. Furthermore, the involvement of lncRNAs in biological processes such as autophagy, apoptosis, adipogenesis, cell proliferation, and cellular differentiation contributes to our comprehension of their roles in the pathogenesis of AS. Nevertheless, the specific mechanisms remain elusive, primarily owing to the scarcity of samples or the intricate interactions with numerous other molecules.

**TABLE 1:** LncRNAs involved in the pathogenesis of AS.

lncRNA	Mechanism	Expression	Source	Type of researches	References
lncRNA H19	inflammatory response	up	human	experimental study	Zhang et al. (2020), Esawy et al. (2023)
lncRNA NKILA	inflammatory response	up	human	experimental study	Vaez et al. (2017), Gai and Li (2019)
lncRNA NONHSAT 227927.1	inflammatory response	up	human	experimental study	Wang et al. (2021), Ding et al. (2023)
lncRNA LOC645166	inflammatory response	down	human	experimental study	Yu et al. (2021)
lncRNA HOTTIP	proliferation of cells	up	mouse	experimental study	Kim et al. (2013), Li et al. (2015), Zhao et al. (2018), Wei et al. (2023)
lncRNA MEG3	differentiation of cells	down	mouse, human	experimental study	Ma et al. (2020), Liu et al. (2023)
lncRNA ZNF354A-1	differentiation of cells	up	human	bio-informatics analysis	Xie et al. (2016)
lncRNA LIN54-1	differentiation of cells	up	human	bio-informatics analysis	Xie et al. (2016)
lncRNA USP50-2	differentiation of cells	up	human	bio-informatics analysis	Xie et al. (2016)
lncRNA FRG2C-3	differentiation of cells	up	human	bio-informatics analysis	Xie et al. (2016)
lncRNA GAS5	autophagy of cells	down	human	experimental study	Tan et al. (2020)
lncRNA HULC	apoptosis of AS cells	up	mouse, human	experimental study	Yi et al. (2023)
lncRNA ENST00000429588.1	adipogenesis of ASMSCs	down	human	bio-informatics analysis	Cen et al. (2022)
lncRNA ENST00000400755.3	adipogenesis of ASMSCs	down	human	bio-informatics analysis	Cen et al. (2022)
lncRNA ENST00000512300.1	adipogenesis of ASMSCs	down	human	bio-informatics analysis	Cen et al. (2022)

AS, ankylosing spondylitis; ASMSCs, ankylosing spondylitis mesenchymal stem cells.

The aforementioned studies underscore the potential of lncRNAs as ideal biomarkers (Table 2) or therapeutic targets. Some of these markers have been confirmed to effectively distinguish patients from controls with considerable sensitivity and specificity. Moreover, many of them exhibit strong correlations with disease severity and prove valuable in predicting the prognosis of AS patients. However, the relevance between the lncRNAs and other indice such as mSASSS and imaging manifestions remain blank for further investigating. From a therapeutic perspective, the reduction of upregulated lncRNAs through RNA interference or CRISPR technology offers novel treatment options for AS, while the augmentation of downregulated ones through gene transfection represents an alternative approach. It can be expected that the application of therapies will be challenging when it comes to the terrible results such as off-target effects and subsequent ethical questions. Only these significant issues were adequately resolved can the clinical use of lncRNAs as therapeutic targets be fully embraced. Furthermore, as evidenced by the tables, many of the researches are bio-informatics analysis, the conclusions of which lack validation. Therefore, additional evidence derived from laboratory experiments, and even clinical experiments, is required to verify specific mechanisms and assess the application value of these findings. Additionally, few included literature differentiated AS into axis- and peri-spondyloarthropathy especially when discussing the mechanisms, so in future researches further classification may help to understand the disease better and treat it more effectively.

**TABLE 2:** LncRNAs involved in the evaluation of AS.

lncRNA	Sample	Expression	Source	Type of researches	References
lncRNA 326C3.7	peripheral blood cells	up	human	experimental study	Wang et al. (2021)
lncRNA-NEF	synovial fluid	up	human	experimental study	Han et al. (2022)
lncRNA TUG1	joint biopsies	down	human	experimental study	Lan et al. (2018)
lncRNA MEG3	joint biopsies	down	human	experimental study	Liu et al. (2019)
lncRNA NONHSAT118801.2	joint biopsies	up	human	bio-informatics analysis	Li and Zhou (2021)
lncRNA NONHSAT183847.1	joint biopsies	up	human	bio-informatics analysis	Li and Zhou (2021)
lncRNA-AK001085	serum	down	human	experimental study	Li et al. (2017)
lncRNA LINC00311	plasma	up	human	experimental study	Zhong and Zhong (2019)
lnc-ITSN1-2	PBMCs	up	human	bio-informatics analysis	Li and Zhou (2021)
lncRNA linc00304	PBMCs	up	human	bio-informatics analysis	Wang et al. (2022)
lncRNA linc00926	PBMCs	up	human	bio-informatics analysis	Wang et al. (2022)

AS, ankylosing spondylitis; PBMCs, peripheral blood mononuclear cells.
